# Maternal-child co-participation in physical activity-related behaviours: prevalence and cross-sectional associations with mothers and children’s objectively assessed physical activity levels

**DOI:** 10.1186/s12889-017-4418-1

**Published:** 2017-05-25

**Authors:** Jill A. Hnatiuk, Ellen DeDecker, Kylie D. Hesketh, Greet Cardon

**Affiliations:** 1 Institute for Physical Activity and Nutrition (IPAN), School of Exercise and Nutrition Sciences, Deakin University, Geelong, Australia; 2 School of Science and Health, Western Sydney University, Locked Bag 1797, Penrith, NSW 2150 Australia; 3 Department of Movement and Sport Sciences, Ghent University, Watersportlaan 2, Ghent, Belgium

**Keywords:** Motor activity, Mother-child relations, Preschool child

## Abstract

**Background:**

Co-participation in physical activity may be a useful strategy for increasing physical activity in mothers and their young children, yet little empirical evidence exists on this topic for young families. This study aimed to identify the prevalence of mother-child co-participation in physical activity and examine the association between co-participatory behaviours and objectively-assessed physical activity in young children and their mothers.

**Methods:**

One-hundred twenty-three 4–6 year-old children and their mothers were recruited from preschools in Belgium between November 2010 and January 2011. Mothers completed a questionnaire assessing the frequency of co-participation in five activities. Both mothers and children wore ActiGraph GT1M accelerometers concurrently for 7 days to assess the time spent in moderate-to vigorous-intensity physical activity (MVPA) and light- to vigorous-intensity physical activity (LMVPA). Descriptive statistics (means, frequencies) were used to determine the prevalence of co-participation. Separate multiple linear regression analyses examined the association between co-participation and mothers’ and children’s physical activity on weekdays and weekends.

**Results:**

Most mothers reported infrequent co-participation in physical activities with their children. On weekdays, walking or cycling for short trips was positively associated with children’s MVPA while attending a park or similar more than once per week was negatively associated with children’s MVPA and LMVPA. Going to an indoor play centre together once or more per week was negatively associated with mother’s LMVPA. On weekends, walking or cycling with their child in their free time was positively associated with both children’s and mothers’ MVPA and childrens’ LMVPA. Going to an indoor play centre together 1–3 times/month was negatively associated with children’s weekend MVPA.

**Conclusions:**

Reported rates of co-participation in mothers and their preschool children were low. The association with maternal and child physical activity may be dependent on the co-participatory behaviour assessed and may differ between weekday and weekends. Promoting walking and cycling together during leisure time may be an effective strategy to increase both preschool children’s and mothers’ MVPA.

## Background

It is well-established that regular participation in physical activity is important for health across the lifespan. Strong evidence exists for the benefits of physical activity on chronic disease risk factors in adults [1] and increasing evidence is emerging in young children [2]. One adult demographic group previously identified as at risk of low physical activity levels is parents of young children (<6 years) [3]. This is particularly evident among mothers of young children, with research suggesting they are 69% less likely to meet physical activity recommendations than adults without dependent children [4]. Although the literature is mixed at present with regard to the proportion of young children who are sufficiently active [5], the high prevalence of inactivity by the time children reach primary school [6, 7] and the progressive decline that occurs in late childhood and adolescence, [8] suggests that the promotion of physical activity in the early years is desirable.

Given this, there is increasing interest in identifying efficient and effective strategies to increase physical activity amongst multiple family members [9]. One possible strategy to increase physical activity in both parents and children is through parent-child participation in physical activity together (co-participation). Based on the social-cognitive theory family perspective [10] it can by hypothesised that co-participation in physical activity be beneficial for physical activity levels of both parents and children through role modelling of the behaviour (predominantly from parents to children) and/or employing reciprocal reinforcement from both parties to foster active pursuits (parents facilitating children’s physical activity and vice versa). This reciprocal reinforcement may also increase the likelihood of continuity of the behaviour within the family over time [11].

For families with young children, co-participation would predominantly occur through co-engagement in active play, active transport or sport. However, there is little evidence on the prevalence of co-participation in the population as well as how it is related to objectively-measured physical activity levels. Several studies involving older children have found that parents and children participate in some physical activity together during the week [12–14] and that parent-child [14–16] or family [15, 17] co-participation in physical activity is associated with higher child physical activity levels across the week. Most research conducted with preschool aged children and their parents have focused on associations between parent/child physical activity [18–20] but haven’t specifically examined whether this physical activity was performed together. Additionally, nearly all studies examining co-participation have focused on the influence that parent-child co-participation has on children’s physical activity levels. Whilst this is important, understanding the role that co-participation has on parents’ physical activity is warranted, as it could assist parents, particularly mothers of young children (a group traditionally at risk of low physical activity levels [4, 21]), to meet physical activity recommendations. Given that co-participation in physical activity is greatest in families when children are in the early childhood years [22], the role of this behaviour may be particularly important during this period of time. Therefore, the aims of the present study were to: 1) examine the prevalence of co-participation in mother-child pairs, and 2) determine the association between co-participation and mothers’ and young children’s physical activity levels.

## Methods

Mothers and children were recruited from preschools in East-Flanders, Belgium between November 2010 and January 2011 as part of a broader study. Principals from 20 preschools were contacted by telephone; all agreed for their school to take part. A letter and informed consent form was then sent home with all children. Mothers of 188 4–6 year-old children provided written consent to take part in the project (response rate: 34%). Research staff visited the preschools, fitted children with accelerometers and provided an envelope for the child to take home to their mother (containing the mothers’ accelerometer, accelerometer instruction sheet, and a questionnaire). Ethics approval for this study was obtained by the Ethics Committee of the University Hospital of Ghent (EC/2006/476).

The child’s mother completed a questionnaire regarding demographic and physical activity-related co-participation behaviours. The demographic variables assessed included: child age (determined by child’s date of birth); child sex; whether the child was attending preschool full-time, maternal age (determined by mothers’ date of birth), education (low = secondary school or less; medium = vocational trade/certificate; high = university degree or higher) and BMI [23], number of siblings in the household (open-ended response) and language predominantly spoken at home (Dutch vs. other).

Questions assessing maternal-child co-participation were purpose-designed and included the frequency of: walking or cycling with their child in their free time (1 = never, 5 = always), playing sport with their child (1 = never, 5 = always) going to the park, playground, beach or similar with their child (1 = never; 10= >5 days per week), going to indoor recreation centre with their child (1 = never; 10= >5 days per week). It also assessed the mode of transport used for short trips (<1 km) with their child (public transport, car, walk, cycle). Due to few responses in extreme categories, those activities scored on a 5-point scale were combined into three groups: never/seldom (1–2), sometimes (3) and often/always (4–5). Activities scored on a 10-point scale were also combined into 3 groups: < once per month (1–5); 1–3 times per month (6–7); ≥ once per week (8–10). The mode of transport for short trips was dichotomized into active (walking, cycling) and inactive (car) as no participants indicated that they mainly used public transport for short trips.

Children’s physical activity was assessed every 15-s using ActiGraph GT1M accelerometers. Children were instructed to wear the activity monitors during all waking hours for seven consecutive days, removing only for sleeping and water-based activities. Twenty-minutes of consecutive zero counts were considered non-wear. Children were included in the final analyses if they had a minimum wear time of 6 h/day for at least 3 weekdays and 1 weekend day [24]. The average daily time spent in weekday and weekend day LMVPA and MVPA was determined by applying Pate cut-points (LMVPA= >35 counts/15 s; MVPA ≥420 counts/15 s) [25]. These two intensities were examined since children’s age at the time of data collection spanned two different sets of physical activity recommendations [26, 27] and because light-intensity physical activity may be a common intensity when parents are engaging in physical activity with their young children [28]. Weekdays and weekend days were examined separately as nearly all children of this age in Belgium attend preschool full time (e.g. 8 am–4 pm) [29]. Thus the opportunity for maternal-child co-participation in physical activity would potentially be greater on weekends compared to weekdays.

Mothers’ physical activity was assessed every 60-s using ActiGraph GT1M accelerometers during waking hours over the same days that their child wore the monitors. Twenty-minutes of consecutive zero counts were considered non-wear. Freedson cut-points [30] were applied to the data to determine the average daily time spent in MVPA during weekdays and weekend days separately (≥10 h/day). Mothers were only included in the final analyses if they wore the monitors for at least 3 weekdays and 1 weekend day [31].

Descriptive statistics (means, frequencies) were used to determine the prevalence of co-participation in this sample and Pearson’s correlation coefficient examined the association between mothers’ and children’s physical activity. Separate multivariate linear regression analyses assessed the association between the five co-participatory activities and mothers’ and children’s weekday and weekend day MVPA and LMVPA. Regression analyses outcome variables are expressed in minutes/day and were adjusted for mothers’ or children’s accelerometer wear time during the weekday/weekend day, sex of the child, maternal education and clustering by preschool the child attended. Data were analysed using Stata 12 [32].

## Results

From the original sample, 51 participants (28%) did not have sufficient accelerometry data (*n* = 6 mothers with incomplete data; *n* = 45 children with incomplete data) and a further 12 (6%) did not have complete questionnaire data. Additionally, two fathers filled out the questionnaires and thus were excluded from these analyses. This left a total of 123 (66%) mother-child pairs to be included in the analyses. Table 1 outlines demographic characteristics of participants. On average, children were just over 5.4 years of age and 50% were boys. All children met physical activity recommendations of at least 60 min MVPA/day [33] when their physical activity level was averaged across all valid days monitored, but only 24% did so when their physical activity was assessed against guidelines for every day monitored. Mothers were highly educated (74% with university degree or higher) and 36% met the adult physical activity recommendations [34]. Mothers and children’s weekday and weekend MVPA was significantly correlated (*r* = 0.23, *p* < 0.05 weekday; *r* = 0.18 *p* < 0.05 weekend) however their weekday and weekend day LMVPA was not.

**Table 1:** Demographic characteristics and objectively assessed physical activity levels of children and mothers

Participant characteristic	
Children	
Age (years)	5.4 (0.32)
Child sex (% male)	50.0%
Attending preschool full-time (5 days/week)	100.0%
Presence of siblings in home (% yes)	88.6%
Predominant language spoken at home (% Dutch)	97.6%
Accelerometer wear time (mins/day)	
Weekdays	701.8 (57.7)
Weekends	678.7 (91.6)
Physical activity (mins/day)	
Weekday MVPA	85.2 (26.4)
Weekend MVPA	81.0 (32.7)
Weekday LMVPA	359.1 (52.7)
Weekend LMVPA	345.3 (67.7)
Proportion meeting children’s physical activity recommendations (60 mins/day on average day)	100.0%
Proportion meeting children’s physical activity recommendations (60 mins/day everyday)	24.0%
Mothers	
Age (years)	34.9 (3.7)
Body mass index	
Underweight (<18.5 kg/m^2^)	5.38%
Healthy (18.5–24.9 kg/m^2^)	72.3%
Overweight (25.0–29.9 kg/m^2^)	17.7%
Obese (>30 kg/m^2^)	4.6%
Education	
Low (Secondary school or less)	2.4%
Mid (vocational trade/certificate)	23.6%
High (university degree or higher)	74.0%
Accelerometer wear time (mins/day)	
Weekdays	901.4 (84.5)
Weekends	832.4 (88.9)
Physical activity (mins/day)	
Weekday MVPA	30.5 (22.9)
Weekend MVPA	25.3 (21.7)
Weekday LMVPA	380.0 (93.9)
Weekend LMVPA	368.9 (93.5)
Proportion meeting adult physical activity recommendations per week (150 mins + MVPA per week)	35.8%

Table 2 outlines the prevalence of mother-child co-participation in the five activities assessed and the associations between co-participation and children’s MVPA and LMVPA on weekdays and weekend days. For most co-participatory variables, few (8–15%) mothers reported engaging in the activities with their child ‘often/always’ or ‘greater than once per week’. However, the majority of mothers (65%) reported using active transport for short trips with their child. On weekdays, attending a park, playground, beach or similar more than once per week was negatively associated with children’s MVPA (β = −19.79 [−30.40, −9.18]) and LMVPA (β = −29.85 [−58.97, −0.73]), while walking or cycling for short trips was positively associated with children’s MVPA (β = 15.73 [6.48, 24.99]). On weekends, walking or cycling with their child in their free time ‘sometimes’ or ‘often/always’ was positively associated with children’s MVPA (β = 9.60 [0.96, 18.24] ‘Sometimes’; β = 15.77 ([3.54, 28.00] ‘Often/always’) and LMVPA (β = 24.09 [9.41, 38.78] ‘Sometimes’). Additionally, going to an indoor play centre together 1–3 times/month was negatively associated with children’s weekend MVPA (β = −17.70 [−29.15, −6.24]).

**Table 2:** Prevalence of maternal-child co-participation and associations with children’s LMVPA and MVPA on weekdays and weekend days^*^

Co-participatory activity	Prevalence N (%)	Children’s Weekday MVPA β (95%CI)	Children’s Weekday LMVPA β (95%CI)	Children’s Weekend MVPA β (95%CI)	Children’s Weekend LMVPA β (95%CI)
Walking or cycling with their child in their free time					
Never/seldom	41 (33%)	Ref.	Ref.	Ref.	Ref.
Sometimes	72 (59%)	3.04 (−6.26, 12.37)	6.21 (−12.66, 25.08)	**9.60 (0.96, 18.24)**	**24.09 (9.41, 38.78)**
Often/always	10 (8%)	5.43 (−7.19, 18.04)	8.26 (−20.73, 37.25)	**15.77 (3.54, 28.0)**	16.68 (−9.88, 43.27)
Playing sport with their child					
Never/seldom	52 (42%)	Ref.	Ref.	Ref.	Ref.
Sometimes	57 (46%)	−3.76 (−16.19, 8.66)	−4.21 (−25.6, 17.16)	−9.51 (−22.07, 3.05)	−8.04 (−28.47, 12.39)
Often/always	14 (11%)	−9.07 (−23.56, 5.40)	−15.53 (−44.02, 12.97)	−11.78 (−26.19, 2.63)	−10.45 (−26.18, 5.29)
Going to the park, playground, beach or similar with their child					
< once per month	46 (37%)	Ref.	Ref.	Ref.	Ref.
1–3 times per month	58 (47%)	−8.31 (−19.99, 3.37)	−10.44 (−32.18, 11.30)	5.74 (−9.39, 20.88)	9.30 (−20.62, 39.21)
≥ once per week	19 (15%)	**−19.79 (−30.40, −9.18)**	**−29.85 (−58.97, −0.73)**	−12.04 (−31.61, 7.53)	−27.36 (−68.21, 13.49)
Frequency of going to an indoor recreation centre with their child					
< once per month	60 (49%)	Ref.	Ref.	Ref.	Ref.
1–3 times per month	26 (21%)	1.40 (−10.74, 13.53)	8.55 (−19.80, 36.90)	**−17.70 (−29.15, −6.24)**	−21.79 (−52.93, 9.34)
≥ once per week	37 (30%)	2.16 (−2.71, 7.02)	1.11 (−14.16, 16.40)	−6.91 (−21.78, 7.95)	−12.14 (−34.36, 10.08)
Mode of transport used for short trips (<1 km) with their child					
Inactive	43 (35%)	Ref.	Ref.	Ref.	Ref.
Active	80 (65%)	**15.73 (6.48, 24.99)**	12.64 (−8.58, 33.84)	2.09 (−9.21, 13.40)	−4.53 (−24.02, 14.97)

^*^Multi-variate linear regression models adjusted for maternal education, child’s sex, accelerometer wear time and clustering by preschool attended; Bolded results are significant at *p* < 0.05

Table 3 outlines the associations between co-participation and mothers’ MVPA and LMVPA. Going to an indoor recreation centre with their child more than once per once or more per week was associated with lower maternal weekday LMVPA (β = −48.95 [−81.31, −16.59]) whilst walking or cycling with their child in their free time ‘sometimes’ (β = 8.31 [0.04, 16.58]) or ‘often/always’ (β = 21.29 [8.19, 34.39]) was positively associated with mothers weekend MVPA.

**Table 3:** Associations between maternal-child co-participation and mothers’ MVPA and LMVPA on weekdays and weekend days^*^

Co-participatory activity	Mothers’ Weekday MVPA β (95%CI)	Mothers’ Weekday LMVPA β (95%CI)	Mothers’ Weekend MVPA β (95%CI)	Mothers’ Weekend LMVPA β (95%CI)
Walking or cycling with their child in their free time				
Never/seldom	Ref.	Ref.	Ref.	Ref.
Sometimes	1.23 (−6.75, 9.22)	12.41 (−23.23, 48.04)	**8.31 (0.04, 16.58)**	14.27 (−28.13, 56.68)
Often/always	19.25 (−2.02, 40.52)	47.20 (−20.20, 114.59)	**21.29 (8.19, 34.39)**	49.20 (−13.04, 111.44)
Playing sport with their child				
Never/seldom	Ref.	Ref.	Ref.	Ref.
Sometimes	4.48 (−5.56, 14.53)	3.82 (−33.14, 40.77)	−4.08 (−12.77, 4.61)	−14.75 (−51.12, 21.62)
Often/always	−2.76 (−22.69, 17.18)	14.49 (−50.53, 79.51)	−10.07 (−24.89, 4.76)	−25.04 (−63.23, 13.15)
Going to the park, playground, beach or similar with their child				
< once per month	Ref.	Ref.	Ref.	Ref.
1–3 times per month	−0.88 (−11.73, 9.96)	3.06 (−48.63, 54.75)	0.74 (−10.55, 12.04)	21. 63 (−19.11, 62.38)
≥ once per week	−13.28 (−27.11, 0.55)	−6.03 (−73.28, 60.68)	−7.80 (−19.30, 3.70)	7.34 (−35.91, 50.59)
Frequency of going to an indoor recreation centre with their child				
< once per month	Ref.	Ref.	Ref.	Ref.
1–3 times per month	−0.74 (−13.68, 12.20)	3.94 (−51.22 (59.11)	−4.22 (−12.77, 4.61)	−24.35 (−69.32, 20.61)
≥ once per week	−0.78 (−8.26, 6.69)	**−48.95 (−81.31, −16.59)**	−3.68 (−12.49, 5.13)	−31.49 (−82.21, 19.22)
Mode of transport used for short trips (<1 km) with their child				
Inactive	Ref.	Ref.	Ref.	Ref.
Active	8.05 (−2.08, 18.18)	27.29 (−5.14, 59.27)	4.74 (−3.63, 13.12)	26.87 (−1.23, 54.96)

^*^Multi-variate linear regression models, adjusted for maternal education, accelerometer wear time and clustering by preschool attended; Bolded results are significant at *p* < 0.05

## Discussion

There is increasing interest in identifying strategies to increase physical activity amongst both parents and young children [9, 21]. This was the first study to report the prevalence of maternal-child co-participation in several age-appropriate activities and to examine the association between maternal-child co-participation and mothers’ and their children’s MVPA. The findings from this study are a platform from which we can build our understanding of family physical activity participation and identify strategies that may be effective at concurrently increasing physical activity in young families.

Overall, maternal-child co-participation was low for most of the variables assessed with approximately 1/3 to 1/2 of participants reporting that they ‘never’ or ‘seldom’ participated in the activity with their child, or did the activity less than once per month. This is consistent with other research which suggests parents and primary school-aged children engage in more sedentary behaviours together compared to active ones [12], and highlights a potential opportunity to promote co-participation to families with young children. The only exception among the variables assessed was in the use of active transport for short trips. The majority (65%) of mothers reported walking or cycling with their child was the main form of transport for distances less than 1 km. While this is comparable to that found in British families [35], it is likely greater than what would be observed in other countries [36]. Nonetheless, the higher prevalence of this co-participatory activity compared to the others may suggest that walking or cycling for short trips is perceived to be feasible by both mothers and children. Thus it may hold potential for incorporating into family-based intervention programs.

A novel aspect of this work was that it examined associations between co-participation and both mothers’ and their children’s MVPA, which may be important for reciprocal reinforcement of health behaviours within families [37]. Only walking or cycling together in their free time was associated with higher MVPA in both parties, and this occurred on weekends only. Given that most children in Belgium attend preschool full time [29], mothers and children may have more time during weekends to engage in this behaviour compared to weekdays. Regardless, given the association observed here, family-based intervention programs may consider specifically incorporating leisure time walking/cycling as a strategy to increase weekend MVPA in both mothers and their children. However, given only one common co-participatory behaviour was observed, a diverse range of co-participatory behaviours, including those not assessed in this study, might need to be promoted to families to effectively increase physical activity in both parties.

Walking or cycling for short trips was positively associated with children’s weekday MVPA. Active travel has previously been associated with greater physical activity in children [35], and as children in the current sample all attended preschool full-time, it is possible that much of the active travel for short trips occurred as active transport to preschool. Thus, efforts to promote active transport to preschool and provide supportive infrastructure to make this feasible for families may increase MVPA in children. However, contrary to hypotheses, frequently going to the park or similar together on weekdays and occasionally going to an indoor play centre together on weekends was associated with lower MVPA in children, inconsistent with previous research [38]. As some research has found that the presence of a parent/adult was associated with lower physical activity amongst children in parks [39], it is possible that depending on the context and interaction, parents can actually reduce children’s physical activity. Thus, understanding the nature and context of the co-participation between parents and children may be crucial. Additionally, frequently attending an indoor play centre together on weekdays was associated with lower LMVPA in mothers. Whilst it isn’t clear why this association was observed, it is possibly that by taking their child to the centre, mothers may actually be replacing time where they might otherwise have been active.

Although this study provides a novel examination of mother-child co-participation in physical activity, several limitations must be acknowledged. First, only five purpose-designed co-participatory variables were assessed. Although these variables capture the main forms of physical activity undertaken for preschool aged children, it is possible that other co-participatory activities not assessed in this study may be associated with mothers’ or children’s MVPA. Additionally, the measures used were purpose designed given the lack of co-participation measures available at the time of data collection. This may have resulted in over- or under-estimation of true co-participation as mothers were not specifically asked to consider only the time that they were actively participating with their child. It is recommended that a comprehensive evaluation of co-participatory measures is developed and validated for use in future research. Finally, the sample was limited in size, cross-sectional in nature and largely relied on a convenience sample of tertiary educated mothers. Thus, investigations of parent-child co-participation should be replicated in a larger, diverse sample, include fathers and utilise longitudinal designs.

## Conclusions

In summary, this study was the first to describe co-participation in physical activity among mothers and their preschool children and associations with mother and child physical activity. Based on the findings, opportunities exist to increase co-participation in mother-child pairs. Additionally, the association between co-participation and physical activity of mothers and children may be activity-specific and differ on weekdays compared to weekends. However, walking or cycling in leisure time might be a promising strategy to increase physical activity of both family members. The findings from this study are a platform from which we can build our understanding of family-based physical activity and identify strategies that can be used in intervention programs to concurrently increase physical activity in mothers and young children.
